# Phytoremediation of heavy metals (Ni, Cd, Pb) by *Azolla filiculoides* from aqueous solution: A dataset

**DOI:** 10.1016/j.dib.2018.10.111

**Published:** 2018-10-28

**Authors:** Dariush Naghipour, Seyed Davoud Ashrafi, Mozhgan Gholamzadeh, Kamran Taghavi, Mohammad Naimi-Joubani

**Affiliations:** a Research Center of Health and Environment, Guilan University of Medical Sciences, Rasht, Iran; b School of Health, Guilan University of Medical Sciences, Rasht, Iran; c Department of Analytical Chemistry, University of Guilan, University Campus 2, Rasht, Iran

**Keywords:** Phytoremediation, *Azolla filiculoides*, Aqueous solution, Heavy metals

## Abstract

In this article, the data of heavy metals phytoremediation efficiency were provided. The *Azolla* was collected from the lake around the Rasht city and washed in tap water, then weighed (0.2, 0.4 and 0.8 g), and kept for 15 days in the 100 ml disposable container in the presence 5, 10 and 25 mg/L of lead, nickel and cadmium ions. The samples were stored in polyethylene containers for analysis of the metal concentration with ICP-OES. According to the results, removal efficiency was increased from 40% to 70% at 10 days along with the increasing of the biomass from 2.0 to 8.0 g. The removal efficiency of Ni (II), Cd (II), and Pb (II) were increased by increasing the contact time up to 10 days. The removal efficiency decreased by increasing of the metals concentration from 5 to 25 mg/L. The highest removal efficiency was observed at heavy metals concentrations of 5 mg/L and contact time of 10 days. Results showed that Azolla had a high potential for the removal of heavy metals from water resources and it can be used in phytoremediation of heavy metals in environmental refinement projects.

**Specification table**

**Table:** 

**Subject area**	Environmental Sciences
**More specific subject area**	Phytoremediation
**Type of data**	Figure and table
**How data was acquired**	Lead, Nickel and Cadmium ions concentration were measured by ICP-OES (Spectro Amitec)
**Data format**	Raw, analyzed
**Experimental factors**	Different concentration of Heavy Metals Lead, Nickel and Cadmium ions in a batch system with different mass of live *Azolla filiculoides* in different retention time was used for the determination of Removal Efficiency
**Experimental features**	Scale up of Phytoremediation results of heavy metals in Lab scale with *Azolla filiculoides*
**Data source location**	Department of Environmental Health engineering, School of Health, Guilan University of Medical Sciences, Rasht, Iran.
**Data accessibility**	Data are included in this article and supplementary file excel.
**Related research Article**	[1], [2], [3], [4], [5], [6].

**Value of the data**

• This data suggest the phytoremediation technique by using the *Azolla filiculoides* to removal of heavy metals in wastewater and natural pools.
• This data can develop the biotechnological methods for large scale wastewater treatment plants.
• This data can be useful for the engineers to design the biological wastewater treatment plants.

## 1 Data

The data of this paper showed the removal efficiency of the *Azolla filiculoides* biomass for lead, nickel and cadmium ions. The range of Ni(II), Cd(II), and Pb(II) removal efficiency using of the *A. filiculoides* biomass were shown in Table 1, Table 2, Table 3, Table 4, Table 5, Table 6, Table 7, Table 8, Table 9 at different conditions. *A. filiculoides* collected from the lake around the Rasht City and some important parameters including; live biomass, contact time, lead, nickel and cadmium concentrations were examined. The highest removal efficiency for cadmium were 92.84%, under biomass of 0.8 g, contact time 15 days, and initial metal concentration of 5 mg/L. The highest removal efficiency for Lead were 97.12%, under biomass of 0.8 g, contact time 15 days, and initial metal concentration of 10 mg/L. The highest removal efficiency for Nickel were 76.82%, under biomass of 0.8 g, contact time 15 days, and initial metal concentration of 25 mg/L.

**Table 1:** Removal efficiency (%) of cadmium (5 mg/L) by *Azolla filiculoides* biomass.

Contact time (day)	Biomass of *Azolla filiculoides* (g)		
	0.2	0.4	0.8
5	64.66	82.33	89.45
10	65.24	85.12	92.11
15	68.04	89.64	92.84

**Table 2:** Removal efficiency (%) of cadmium (10 mg/L) by *Azolla filiculoides* biomass.

Contact time (day)	Biomass of *Azolla filiculoides* (g)		
	0.2	0.4	0.8
5	40.33	68.82	70.11
10	41.95	69.67	72.2
15	46.78	75.22	89.1

**Table 3:** Removal efficiency (%) of cadmium (25 mg/L) by *Azolla filiculoides* biomass.

Contact time (day)	Biomass of *Azolla filiculoides* (g)		
	0.2	0.4	0.8
5	34.1	40.33	83.23
10	36.32	45.04	78.14
15	38.21	74.69	66.15

**Table 4:** Removal efficiency (%) of nickel (5 mg/L) by *Azolla filiculoides* biomass.

Contact time (day)	Biomass of *Azolla filiculoides* (g)		
	0.2	0.4	0.8
5	42.33	57.41	68.87
10	46.54	61.48	69.85
15	49.67	53.36	71.12

**Table 5:** Removal efficiency (%) of nickel (10 mg L^−^^1^) by *Azolla filiculoides* biomass.

Contact time (day)	Biomass of *Azolla filiculoides* (g)		
	0.2	0.4	0.8
5	41.34	51.7	56.3
10	43.61	63.23	63.33
15	45.13	57.74	69.42

**Table 6:** Removal efficiency (%) of nickel (25 mg/L) by *Azolla filiculoides* biomass.

Contact time (day)	Biomass of *Azolla filiculoides* (g)		
	0.2	0.4	0.8
5	35.56	48.77	53.34
10	37.94	47.52	52.44
15	39.65	44	76.82

**Table 7:** Removal efficiency (%) of lead (5 mg/L) by *Azolla filiculoides* biomass.

Contact time (day)	Biomass of *Azolla filiculoides* (g)		
	0.2	0.4	0.8
5	59.76	72.31	89.87
10	60.9	60.9	91.25
15	62.7	62.7	95.41

**Table 8:** Removal efficiency (%) of lead (10 mg/L) by *Azolla filiculoides* biomass.

Contact time (day)	Biomass of *Azolla filiculoides* (g)		
	0.2	0.4	0.8
5	30.21	60.46	68.55
10	36.94	36.94	77.84
15	41.1	41.1	97.12

**Table 9:** Removal efficiency (%) of lead (25 mg/L) by *Azolla filiculoides* biomass.

Contact time (day)	Biomass of *Azolla filiculoides* (g)		
	0.2	0.4	0.8
5	23.1	56.5	75.68
10	25.89	25.89	77.16
15	37.55	37.55	79.24

## 2 Experimental design, materials and methods

### 2.1 Materials

All chemicals used in this experiment were analytical grade and purchased from Sigma-Aldrich.

#### 2.1.1 Preparation of metals stock solutions

The metals stock solution of Ni(II), Cd(II), and Pb(II) with initial concentration of 1000 mg/L were prepared by dissolving an appropriate amount of nitrate salts of these metals(i.e., Ni(NO_3_)_2_·6H_2_O, Cd(NO_3_)_2_·4H_2_O, and Pb(NO_3_)_2_, respectively) in double distilled water. To provide the heavy metals solution with concentrations of 5,10 and 25 mg/L, the stock solution was diluted by using double distilled water.

### 2.2 Experimental procedures and methods

The fresh *A. filiculoides*, used here were collected from the lake around the Rasht City and washed with urban water and sterilized with Mercuric chloride (0.1%) for 30 s, then washed with deionized water several times [7], [8], [9], [10]. Some of fresh Azolla was plotted between two sheets of tissue paper and air dried about 30 min, afterwards weighed in (0.2, 0.4 and 0.8 g) and put them in the cylindrical plastic dishes with 9 cm depth and approximately 200 cm^3^ volumes and filled with 100 ml of test solutions (5,10 and 25 mg/L) of Ni(II), Cd(II), and Pb(II) and deionized water as a control Inoculum solution.

**Fig. 1:**
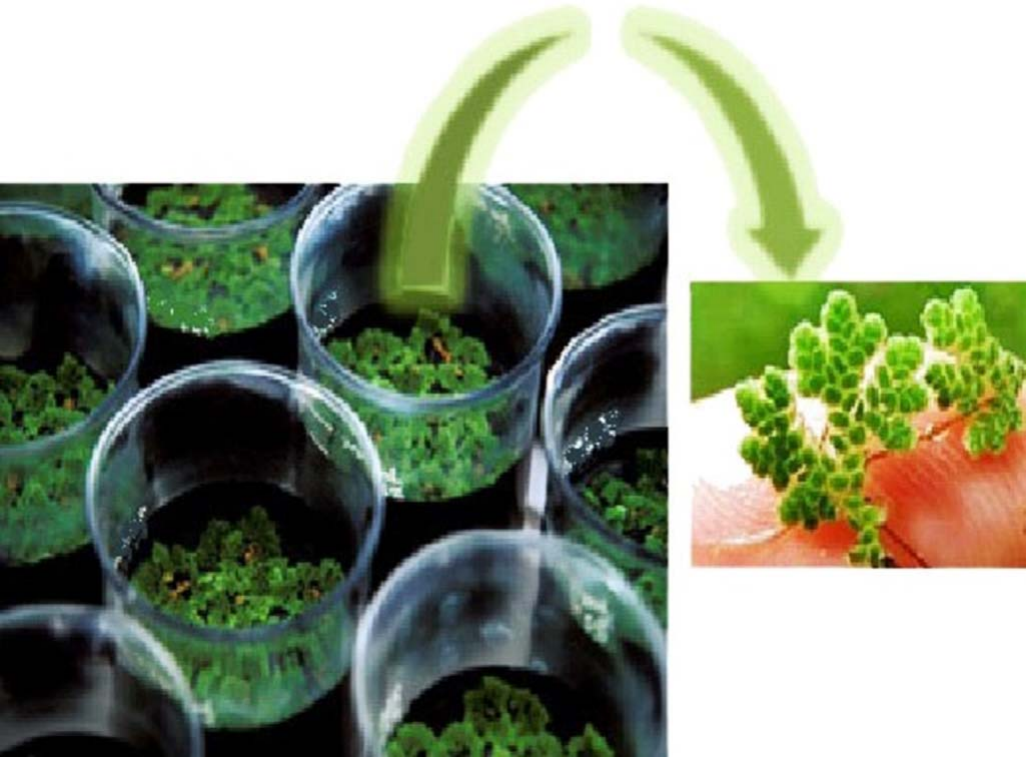
The pictures of the reactor and the used plant in experiments.

All the dishes were kept for 15 days under 40 W fluorescent lamp as a light source and greenhouse conditions. During the incubation the evaporated water was compensated by adding deionized water when needed (Fig. 1).

Sampling was done every five days until the 15th day of the experiment. Water samples were stored in polyethylene containers to read the heavy metals concentration with ICP-OES [11], [12], [13], [14], [15]. All experiments were conducted triplicates and the data were statistically analyzed by Microsoft Excel 2010.

The data of heavy metals concentration in the Phytoremediation process [16], [17], [18] were collected and the removal efficiency percentage was calculated by the following equation (Eq. (1));

(1) $$\mathrm{Removal}\;\mathrm{efficiency}\;(\%)=100\times \frac{C_{0}-C_{t}}{C_{0}}$$

where *C_t_* is the concentration (mg/L) at the end of adsorption and *C*_0_ is the initial concentration (mg/L) of heavy metals.

## Figures and Tables

**Fig. S1:**
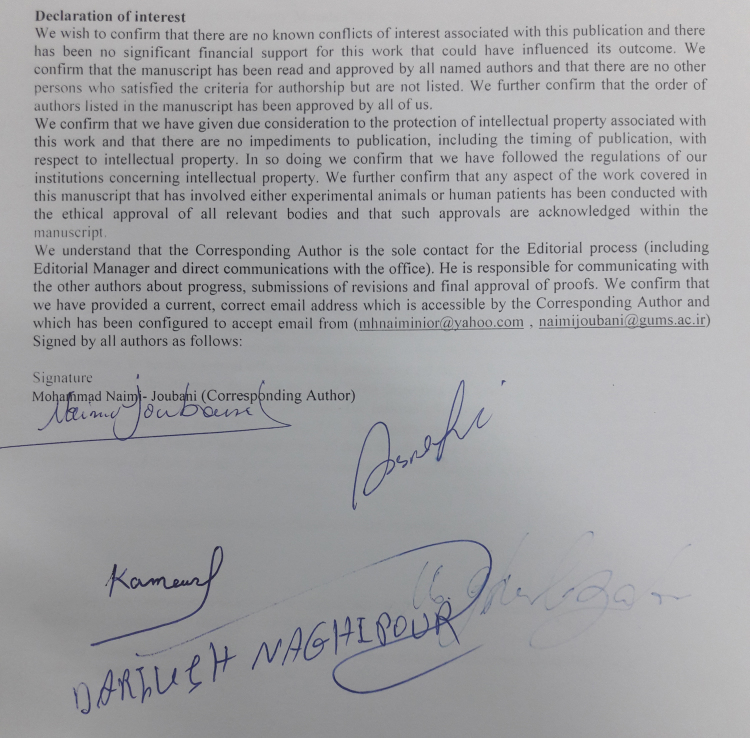
The core spectra of the chips’ surfaces after APTES and GO-COOH coating.
